# Achieving cell-type selectivity in metabolic oligosaccharide engineering

**DOI:** 10.1039/d5cb00168d

**Published:** 2025-07-29

**Authors:** Michelle Marie B. Helmeke, Rhianna L. Haynie-Cion, Matthew R. Pratt

**Affiliations:** a Department of Chemistry, University of Southern California Los Angeles California 90089 USA matthew.pratt@usc.edu

## Abstract

Metabolic oligosaccharide engineering (MOE) is a transformative technology, enabling the chemical labeling and subsequent analysis of glycans. Central to this method are monosaccharide analogs, termed metabolic chemical reporters (MCRs), that contain abiotic functional groups that can undergo an increasing number of bioorthogonal reactions. Typically, these abiotic groups were designed to be as small as possible, allowing them to be tolerated by metabolic enzymes and glycosyltransferases that transform MCRs into donor sugars and add them into glycans, respectively. This generality allows MCRs to be used by a variety of cells and tissues but can also be a limitation in their application to investigate glycosylation of specific cell-types in multicellular systems. Here, we review different methods that are beginning to transition MCRs into cell selective tools, with the potential to increase the already large impact these compounds have had on glycoscience.

## Abstract 2

Metabolic oligosaccharide engineering (MOE) is a transformative technology, enabling the chemical labeling and subsequent analysis of glycans.

## Graphical abstract

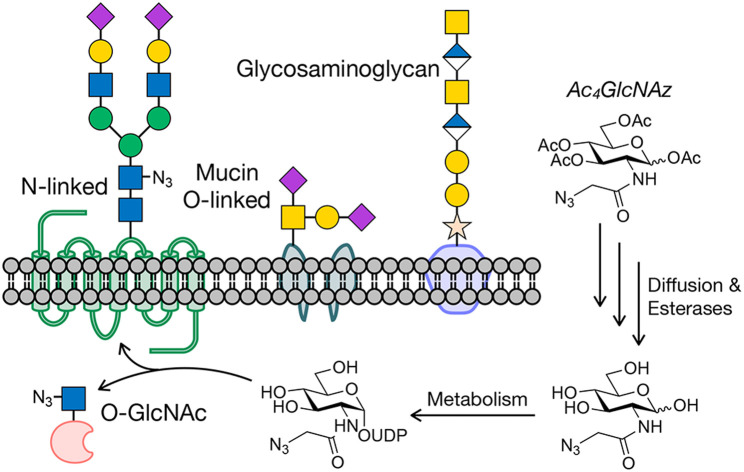

## Introduction

**Fig. 1:**
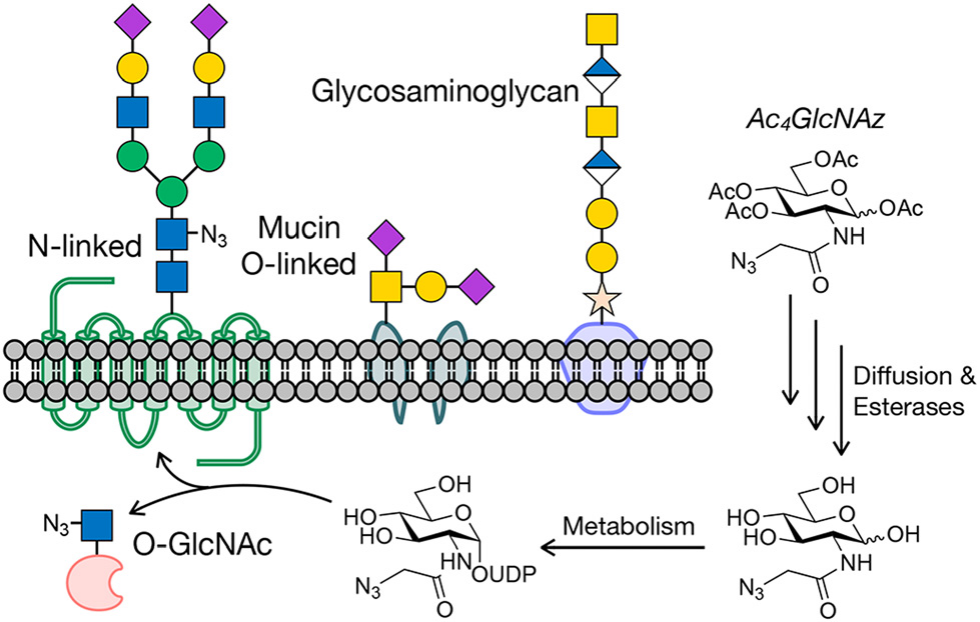
Protein glycosylation and metabolic oligosaccharide engineering. (a) Glycosylation occurs on cell surface and secreted proteins, as well as intracellular proteins. Metabolic oligosaccharide engineering (MOE) takes advantage of metabolic chemical reporters (MCRs) bearing bioorthogonal handles, like Ac_4_GlcNAz, that can be fed to cells where they are metabolized to donor sugars and incorporated into glycosylation.

The addition of carbohydrates to proteins is an abundant posttranslational modification.^1–3^ In animals, most glycosylated proteins, termed glycoproteins, are found on the cell surface or on secreted proteins. These glycans are typically oligosaccharides made up of diverse patterns of individual monosaccharides, generating potentially large levels of structural diversity even on the same underlying protein. The major classes of extracellular glycosylation include N-linked glycosylation, mucin O-linked glycosylation, and proteoglycans (Fig. 1). N-Linked glycans play critical roles in protein folding in the endoplasmic reticulum and trafficking of proteins to the lysosome.^4–7^ N-Linked glycans can also serve as ligands for binding proteins, control the half-lives of secreted proteins, and alter effector functions of antibodies. Mucin O-linked glycosylation is named after the mucin glycoprotein, which is heavily glycosylated and serves as lubricants and protective barriers.^8,9^ These glycans can also change the stability of secreted cytokines and serve as protein ligands. Intracellular proteins can also be dynamically glycosylated by a single monosaccharide. Proteoglycans are long polymers of repeating disaccharide units that are often elaborated by sulfation at multiple positions, setting up a wide array of possible sulfation patterns, which define the binding of GAGs to different proteins.^10–12^ GAG–protein interactions are fundamental to many biological pathways, including growth factor signaling^12,13^ and axonal homing during brain development and potential regeneration.^14^ Intracellular proteins can also be dynamically modified by a class of glycosylation termed O-GlcNAc modification (Fig. 1).^15–17^ This glycan is not further elaborated and plays several roles in signaling, protein aggregation, transcriptional regulation, *etc.*

Tools that enable the enrichment and identification of glycoproteins bearing different classes of glycans are critical for investigating the underlying biochemistry that explains these biological associations. Unfortunately, traditional biological reagents fall short in their ability to act as robust and/or selective detection or enrichment tools. Antibodies selective for protein targets are relatively easily generated, as most antigens possess unique regions that have not previously been encountered by relevant components of the immune system, particularly when injected into other animals (*e.g.*, mice or rabbits). In contrast, the monosaccharide constituents that make up glycans are universal in mammals and abundant on the cell surface and are therefore largely recognized as self-antigens. Many glycans are comprised of heterogeneous mixtures of complex structures. These features complicate the generation of anti-glycosylation antibodies.^18–20^ Native carbohydrate-binding proteins called lectins can overcome some of these limitations,^21^ but their binding is typically driven by avidity, resulting in weak affinity for enrichment-based applications.

Progress is still being made in the development of both antibody- and lectin-based tools, but chemical approaches have also richly contributed to cataloging glycoproteins. One of the first such methods, termed metabolic oligosaccharide engineering (MOE),^22–24^ was first created by the Bertozzi lab around the turn of the last century.^25,26^ MOE relies on monosaccharide analogs, which we have named metabolic chemical reporters (MCRs), that contain chemical groups that undergo selective bioorthogonal reactions for the installation of affinity tags for enrichment and subsequent proteomics/glycomics. An example of a typical MCR, Ac_4_GlcNAz,^27^ is shown in Fig. 1. Upon treatment of cells or living organisms, Ac_4_GlcNAz will diffuse across the cellular membrane and undergo de-*O*-acetylation by one or more as yet unidentified esterases. Because of the small size of the azide-group at the *N*-acetyl position, GlcNAz can then be metabolized into the corresponding UDP-GlcNAz donor, which can then be used by glycosyltransferases, resulting in incorporation of the azide into glycans. A variety of bioorthogonal reactions, like the copper(i)-catalyzed or strain-promoted azide–alkyne cycloadditions (CuAAC or SPAAC), can then be exploited to install tags.^28^

Several different labs have contributed MCRs based on different monosaccharide scaffolds bearing different bioorthogonal reactive groups.^22–24,28,29^ These MCRs display different glycan-class distributions and have distinct advantages/disadvantages depending on the biological application (proteomics, imaging, *etc.*). Despite these distinctions, traditional MCRs tend to label all types of animal cells due to ubiquitous expression of carbohydrate salvage pathway enzymes for metabolism and glycosyltransferases for incorporation into glycans.^30^ While this property makes MCRs versatile tools, it limits the potential experiments that can be performed *in vivo* or in multicellular systems. For example, tumor cells live and grow in a heterogeneous microenvironment containing multiple other cell types (fibroblasts, immune cells, *etc.*). Likewise, during development neighboring cells commit to different differentiation pathways to enable the organization of organs, *etc.* Given the documented role for glycosylation in mediating various relevant biological pathways,^31^ including cell–cell communication and transcription, MCRs that can catalog the glycoproteins and glycans on specific cells in these environments could illuminate new biology. In this short review, we outline different approaches to create cell-selective MCRs. We begin by discussing strategies to deliver more standard MCRs to certain cells over others by taking advantage of liposomes. We then go on to describe MCRs that cannot be metabolized until strategically-placed “caging” groups are removed and finally chemical-genetic strategies to engineer MCR-enzyme pairs. Finally, we conclude with a discussion of some known limitations of MCRs and the future outlook for chemical biologists and glycoscientists.

## Liposome delivery

**Fig. 2:**
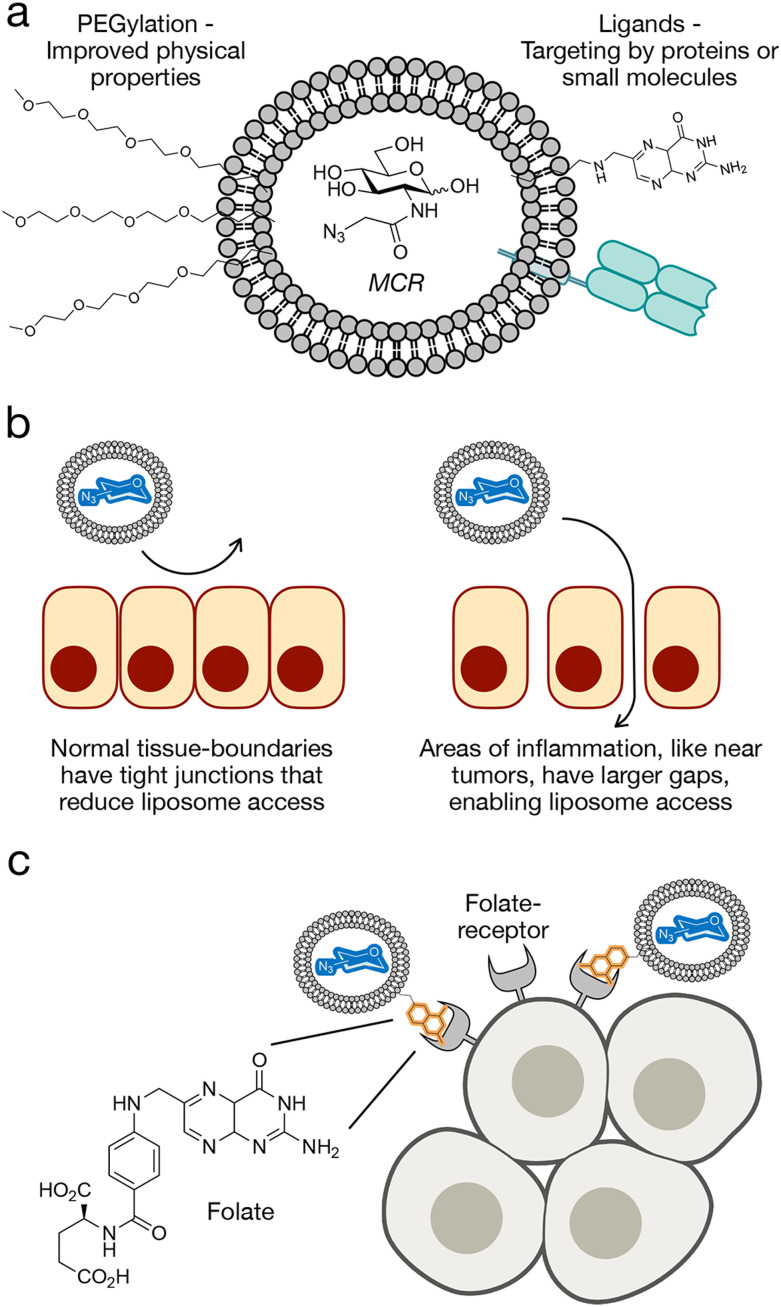
Liposome delivery of MCRs. (a) Liposome features including PEG-chains for improved stability and targeting ligands for cell-selective delivery. (b) Liposomes show inherent selectivity for tumors based on the enhanced permeability and retention (EPR) effect. (c) Targeting of liposomes to folate-receptor expressing cells as an example of cell-selective MCR delivery.

Nanoparticles (NPs) are a drug delivery system that have been recently utilized to deliver MCRs to tissues, and their structure allows them to easily incorporate tissue specific features. Nanoparticles can be synthesized using several different natural, organic, and inorganic materials to generate micelles and liposomes.^32–34^ Liposomes are the most common type of nanocarriers that are composed of one or more lipid bilayers, forming a spherical lipid vesicle (Fig. 2(a)). Additionally, polyethylene glycol (PEG) groups are often attached onto the phospholipids in order to enhance circulation time, reduce immunogenicity, and improve targeting capabilities.^35^ The intended drug molecule can either be loaded into the aqueous hydrophilic core or in the hydrophobic bilayer.

The general size of nanoparticles can be used to achieve target specificity to tumors through the enhanced permeability and retention (EPR) effect (Fig. 2(b)).^36,37^ The EPR effect is a phenomenon in which macromolecules such as nanoparticles accumulate in areas of inflammation such as tumors and cancerous tissues. This property is due to an increase in passive accumulation, as the nanoparticles, especially in the nanometer size can more easily enter tumors due to larger gaps between epithelial tissue and higher permeability. Additionally, cancerous tissues exhibit less lymphatic drainage which prevents the clearance of macromolecules as quickly and results in higher retention and accumulation. Altogether, this phenomenon makes nanoparticle and liposome delivery a useful drug delivery system for tumor targeting. However, since some parts of solid tumors are pathophysiologically heterogeneous, some parts of the tumors are not vascularized, and cannot be targeted using the EPR effect with their varied microvascular permeability.^38^

In an alternative and potentially more specific strategy, nanoparticles and liposomes can also be engineered to have ligands that specifically target a cell surface receptor that may be overexpressed in the target tissue or on the target cell. These ligands can be covalently or noncovalently attached on the liposome surface, but more often they are attached to the distal end of the PEG chain in order to reduce steric hinderance with the PEG groups.^39^ Several targeting strategies can be utilized such as peptides, proteins, antibodies or protein fragments, carbohydrates, nucleic acids, and small molecules.^40–44^ This active targeting system has been able to localize drugs with higher effectiveness, reduce drug dosages, minimize side effects, and reduce drug variation in blood concentration.^45,46^

One challenge with MCR delivery has been the ability to transport MCRs across the blood brain barrier (BBB). This problem is most likely due to the inability of reporters to cross the BBB. The ability to probe sialic acid metabolism in the brain has important applications, as aberrant sialylation has been implicated in cancer cell metastasis to the brain, lysosomal storage disorders, and neurodegenerative diseases.^47–49^ The Chen group was able to harness liposomes and created a liposome-assisted bioorthogonal reporter (LABOR) strategy.^50^ They determined that liposome encapsulated 9-azido sialic acid (AzSia) was not only able to image brain sialoglycans, but also to label distinct mouse brain regions. They were also able to successfully show the utility of their LABOR strategy by doing proteomics analysis of sialylated glycoproteins in the brain. This LABOR strategy has now allowed for azido sugars to be successfully delivered to the brain which can be used for future studies.

In addition to their general biophysical properties, the liposomes can easily incorporate cell specific ligands that can be used to selectively deliver MCRs to certain cell types (Fig. 2(c)). One example of this was the Chen lab who used folate-targeted liposomes (f-LPs) to deliver 9AzSia to folate receptor (FR)-expressing cells.^51^ This could be used to target several epithelial-derived tumors such as ovarian, breast, lung, and colorectal cancers; and this receptor has already been previously used for targeted drug delivery to cancers.^52^ The authors were able to achieve selectivity for HeLa cells overexpressing FR compared to normal HeLa cells. Additionally, they showed that the liposomal carriers without the folic acid targeting ligand resulted in weak or little fluorescence labeling, showing the importance of the targeting ligand.

The same group subsequently demonstrated the viability of ligand targeted liposomes *in vivo* using a different targeting system.^53^ Specifically, they used a cyclic peptide as the targeting ligand to probe sialylated glycans in a xenograft model of melanoma in living mice. The cyclic peptide, Arg-Gly-Asp-d-Tyr-Lys (cRGDyK) pentapeptide is recognized by integrin v3, which is overexpressed on the B16-F10 cell surfaces which is a mouse melanoma cell line. In a xenograft model, their cyclic pentapeptide liposomal carrier was able to selectively label B16-F10 tumor cells while exhibiting minimal fluorescence in a control MCF-7 tumor. Additionally, there was a much weaker signal when testing the liposomal carrier without the cyclic peptide, showing that simply relying on the EPR effect is insufficient for selective labeling in this case. Simple treatment with Ac_4_ManNAz alone also resulted in low tumor targeting efficiency. These results demonstrate that an active targeting mechanism can not only achieve selectivity but also improve the incorporation of azides into the tumor-associated glycans.

The use of the LABOR strategy also allows for simultaneous targeting and delivery of unique MCRs to different cell-types by taking advantage of differential expression of cell surface proteins. Again, the Chen group tested this using a new targeting system for K20 immune cells in combination with their previous folate receptor targeting system.^54^ They treated a co-culture of K20 immune cells and FR overexpressing HeLa cells, with their two different liposomal carriers. They used BPC NeuAC-LP-SiaNAl that targets K20 cells with a glycan ligand and delivers an alkyne functionalized sialic acid and their folate targeting liposome, f-LP-9AzSia, which delivers an azide functionalized sialic acid. By reacting with DBCO-carboxyrhodamine 110 and azide-AF647 for the SiaNAl and 9AzSia, respectively, the K20 and FR+ HeLa cells were successfully distinguishable by flow cytometry. They were able to show that using two different ligand–receptor pairs can successfully label glycans of different target cells with distinct chemical tags.

The delivery system of liposomes has addressed limitations in MCR delivery such as crossing the blood brain barrier and selectivity, and the general properties of nanocarriers can be exploited by other classes of structures like dendrimers.^55^ The process to make liposomes selective is a fast and easy process.^56^ With liposome and nano-carriers, the selective agents can be attached to the PEG group and the MCRs can be easily loaded as cargo. For differing sugar molecules, the same ligand-targeted liposome can be used by encapsulating a different sugar moiety. Overall, the use of liposomes for MCR delivery has proved to be a promising strategy to achieve increased labeling efficiencies *in vivo*, as well as discrimination between cell-types based on known receptor–ligand interactions.

## Caging groups

**Fig. 3:**
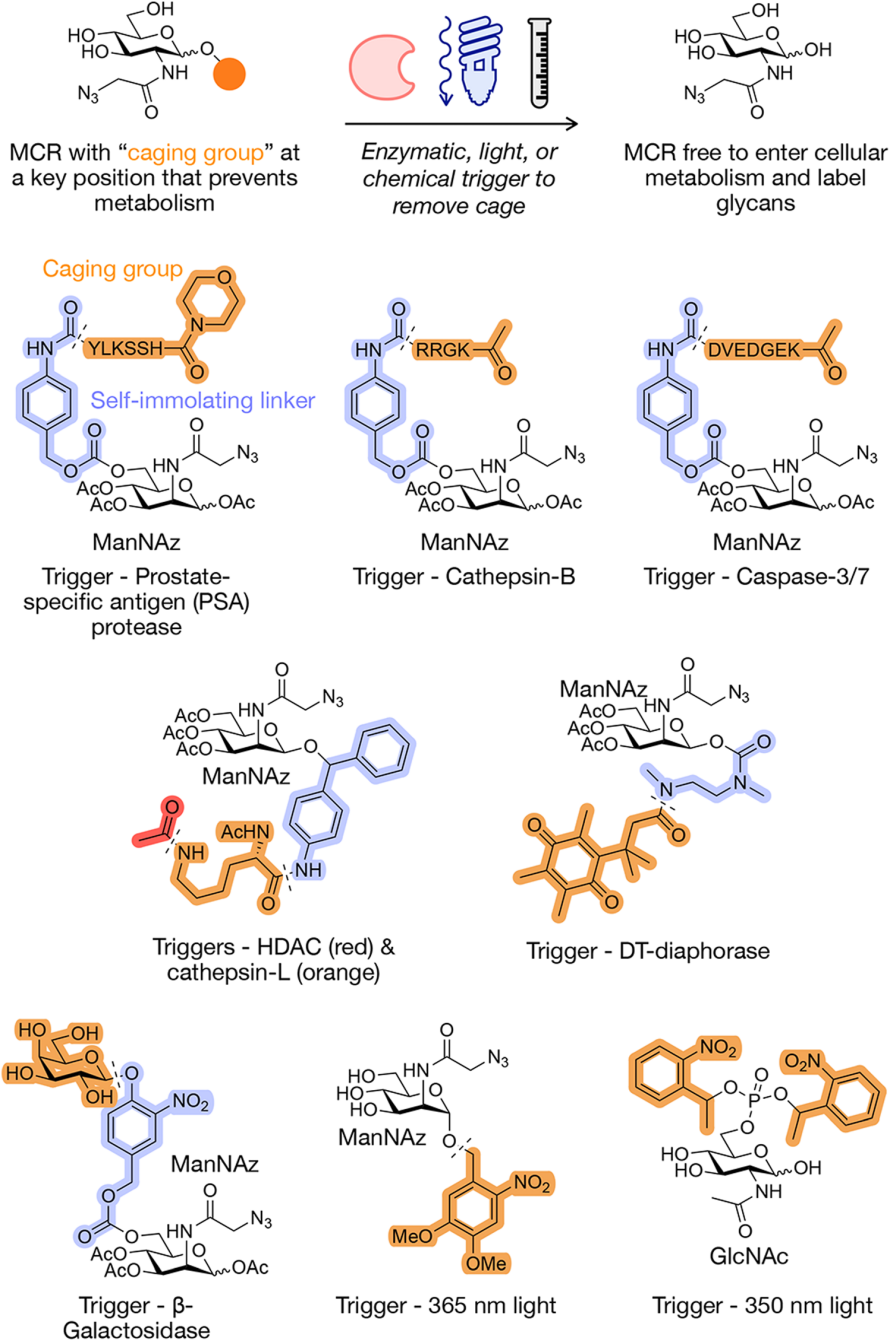
Caged MCRs and monosaccharides. A caged group can be added at a position that prevents a downstream metabolic transformation. Various triggers can be used to remove the cage to enable glycan labeling. Examples of caged MCRs and GlcNAc covered in this review.

Another drug delivery system that has been utilized for selective delivery of MCRs is through the use of caging groups similar to some strategies for pro-drugs (Fig. 3). Pro-drugs are chemically modified versions of an active agent that undergoes a transformation *in vivo* to release the active agent. This can improve physiochemical, biopharmaceutical or pharmacokinetic properties of compounds.^57–61^ Around 5–7% of the drugs worldwide can be classified as prodrugs.^58^ In 2015, prodrugs made up over 15% of the total amount of FDA approved drugs in that year.^62^ One of the earliest prodrugs that was approved by the FDA for use in the US and is still used to treat autoimmune conditions including Crohn's disease, ulcerative colitis, and rheumatoid arthritis is sulfasalazine.^63^ Once sulfasalazine reaches the colon, it is metabolized by bacteria into 5-aminosalicyclic acid (5-ASA) which inhibits the production of inflammatory factors in the gut and sulfapyridine which is unfortunately responsible for some adverse side effects of sulfasalazine. One of the largest limitations of prodrugs is toxicity, and an important part of developing a pro-drug is to use a promoiety or caging group that does not form an undesired metabolite and one that is rapidly excreted from the body. Additionally, the caging group must only be able to be decaged in the presence of the decaging agent to reduce drug delivery to unwanted tissues. While these are some limitations, their ability to help with solubility, chemical instability, insufficient oral absorption, rapid pre-system metabolism, brain penetration, toxicity, and irritation make it a promising drug delivery strategy.

For MCR delivery, the pro-drug delivery strategy has been successfully utilized to solve the issue of cell-selective labeling of specific tissues and organs. In this case a caged functional group prevents the MCR from being incorporated into the metabolic pathways of unwanted cells and tissues, and only cells with the overexpressed decaging agent (*e.g.*, an enzyme) will metabolize the free sugar analog (Fig. 3). The first example of the use of this strategy for MCR delivery was the Bertozzi group who synthesized a variant of Ac_3_ManNAz with a 6-hydroxyl group conjugated through a self-immolating linker to a peptide substrate for the prostrate-specific antigen (PSA) protease.^64^ This protease is secreted at low levels by normal glandular cells, but highly upregulated by prostate cancer cells.^65–67^ It has been previously used as a caging agent in order to target these types of cells. When PSA cleaves its peptide substrate from a *p*-aminobenzyl alcohol linker, the linker will spontaneously fragment and release Ac_3_ManNAz, which can then undergo typical MCR metabolism resulting in azido-sialic acids on the cell surface for further conjugation to fluorophores for imaging *via* bioorthogonal chemistry. They were able to show that labeling intensity is PSA and substrate concentration dependent, the signal is due to PSA activation of the probe, and that this is a relevant and viable option for selectively delivering MCRs into cells for labeling.

One downside to using a PSA decaging strategy is that this method cannot be used *in vivo* as the PSA proteases in the blood will likely degrade the PSA protease-specific MCRs and the uncaged metabolites can be taken up by normal cells, resulting in unspecific labeling. The Kim group wanted to create a caged MCR that could be used *in vivo* to selectively deliver MCRs to various tumor cells and incorporate azides onto the cell surface that could be used as “artificial chemical reporters.”^68^ Creating artificial chemical reporters on the cell surface can be used for further fluorescent tumor imaging as well as nanoparticle targeting, as the expression level of biological receptors can be insufficient for nanoparticle targeting. They synthesized their caged MCR, RR-S-Ac_3_ManNAz, using a cathepsin-B-specific cleavable peptide (Lys-Gly-Arg-Arg, RR) to cage Ac_3_ManNAz with a *p*-aminobenzyloxycarbonyl linker (S). Cathepsin-B is a cysteine protease that is abundant in various tumor cells such as colorectal cancer, malignant glioma, breast cancer, prostate cancer, and melanoma.^69,70^ Their caged MCR showed cathepsin-B selectivity when tested against cathepsin D, E, L, and caspase-3. They were also able to show the applicability of this MCR *in vivo* by showing higher tumor-cell specificity of RR-S-Ac_3_ManNAz for xenograft tumors in a cathepsin-B activity-dependent manner. They were able to successfully generate an exogenous chemical receptor on the surface of tumor cells which can then be further used for tumor-specific targeting or drug delivery.

The same group also utilized their pro-drug strategy to specifically track tumor-cell apoptosis.^71^ The dysregulation of apoptosis, or programmed cell-death, can lead to various diseases such as Alzheimer's, AIDS, autoimmunity, heart disease, and cancer.^72–76^ Methods that can directly visualize and quantify apoptosis in tumor cells can be utilized for predicting anticancer efficacy and optimized the selection of anticancer drugs.^77,78^ Specifically caspase-3 and caspase-7 are cysteine–aspartic acid proteases which can directly execute apoptosis after sequential activation by caspase-8 or caspase-9.^79^ Therefore, caspase activity can be used as a surrogate measurement of apoptosis. The Kim group engineered an MCR that can be used to monitor apoptosis, Apo-S-Ac_3_ManNAz, using a caspase-3/-7 specific cleavable peptide (KGDEVD, Apo) conjugated to Ac_3_ManNAz with a *p*-aminobenzyloxycarbonyl linker (S). The Apo-S-Ac_3_ManNAz exhibited specificity for apoptotic tissues induced by either the tumor necrosis factor-related apoptosis-inducing ligand (TRAIL) or doxorubicin (DOX). Furthermore, fluorescence detection of MCR labeling correlated with DOX concentration, successfully showing that their MCR can be used to image different levels of apoptosis, but can also be used to screen the anticancer ability of potential therapeutics.

The Cheng group extended the development of caged MCRs through a Ac_3_ManNAz derivative that is controlled by two enzymatic activities.^80^ Specifically, they chose histone deacetylase (HDAC) and cathepsin-L activities to yield greater selectivity for cancer cells that overexpress both of these enzymes.^81^ In contrast to the methods described above, the authors chose to move their caging group from the 6-hydroxyl of the MCR to the anomeric position. As a proof of principle, they first caged this position as a UV-cleavable 2-nitrobenzyl ether bond, which they were able to efficiently cleave with light to induce cell surface azido-sialic acids. They then used a self-immolative linker, 4-aminophenyl(phenyl)methanol (PL), linking Ac_3_ManNAz to an acetylated lysine analog. In an ordered decaging HDAC removes the *N*_6_-acetyl group of the lysine residue first, followed by cleavage of the resulting lysine by cathepsin-L. After activity from both enzymes, the self-immolative linker will then undergo structural rearrangement to release the metabolically active Ac_3_ManAzOH. The authors found that this double caging group provided selectivity for tumor tissues with minimal labeling in the liver, kidney, spleen, lung, and heart compared to Ac_4_ManNAz. They were also able the show the applicability of these MCRs for potential drug delivery using a bioorthogonal–doxorubicin-conjugate, resulting in drug accumulation and enhanced anticancer activity in a xenograft model.

Under certain conditions, some self-immolative linkers can become unstable under physiological conditions which can result in cleavage of the caged group in the absence of a trigger. The Cheng group explored this issue by testing the background stability of four different linkers in cells.^82^ They found that an *N*,*N*′-dimethylethylenediamine linker showed negligible labeling in all 6 cell lines. Once they were able to find a linkage that would not decompose and label without a triggering mechanism, they then incorporated a DT-diaphorase responsive element with a trimethyl quinone moiety (HQ). DT-diaphorase is an enzyme that is overexpressed in cancer, and its high activity is associated with hypoxia and cancer cell aggressiveness.^83^ Their HQ caged MCR with the N-carbamate linker was successfully able to respond to endogenous DTD and install azide moieties on the cell surface.

Another example of caged systems that are based on enhanced enzymatic activity is an Ac_3_ManNAz MCR with a caged group consisting of a β-linked galactose monosaccharide and a self-immolating linker, termed Gal-AAM.^84^ The authors found that this caged group could be selectively cleaved by β-galactose which is upregulated in cancer cells. The Gal-AAM MCR was more efficient at selectively labeling tumor cells compared to Ac_3_ManNAz. This study also further exhibited potential applicability for targeted immunotherapy. After treating with Gal-AAM, they took advantage of the resulting cell-surface azides to bioorthogonally install either l-rhamnose or a double zinc finger domain, which could be recognized by antibodies already present in human serum. The authors showed that this double-labeling strategy was able to increase the cytotoxicity against cancer cells from less than 10% for Ac4ManNAc control-treatment groups to 44–49% of MDA-MB-231 and MCF-7 cells killed mediated by rhamnose-specific antibodies or IgG in antibody-dependent or complement-dependent cell death assays.

While enzymes are a popular target for a decaging strategy, it is not the only way decaging can be controlled. Building upon the work using an unsubstituted nitrobenzyl group described above, the Chen group improved upon this strategy using a more reactive 4,5-dimethoxy-2-nitobenzyl (DMNB) group to the anomeric C1 group of ManNAz, again preventing its metabolism.^85^ This MCR was able to successfully decage upon 365 nm light with spatiotemporal control as well as with single-cell resolution, using a less damaging wavelength of light and moving closer to caged groups that might enable *in vivo* applications. For different sugars, there are several points in the metabolic process that could be blocked to create caged MCRs. To explore this possibility, the Fehl group wanted to determine different metabolic intermediates in the GlcNAc, GalNAc, and sialic acid pathways. Specifically, they synthesized compounds that either had a UV-dependent caged group directly at the anomeric position or metabolically-downstream analogs bearing caged sugar–phosphates.^86^ They found that all of these monosaccharides could be decaged and that certain caged-compounds induced cell toxicity, highlighting the importance of selecting the appropriate caged position for this class of MCRs.

The pro-drug, caging strategy for site-specific delivery of MCRs has been successful at achieving specificity through utilizing several different overexpressed enzymes and UV light. The specific decaging and release of the free sugar analog has also allowed for the successful installation of azide groups onto the cell surface of cells which can be further used to investigate the role of metabolic pathways in disease, imaging, and targeting for drug therapies.

## Chemical genetics

**Fig. 4:**
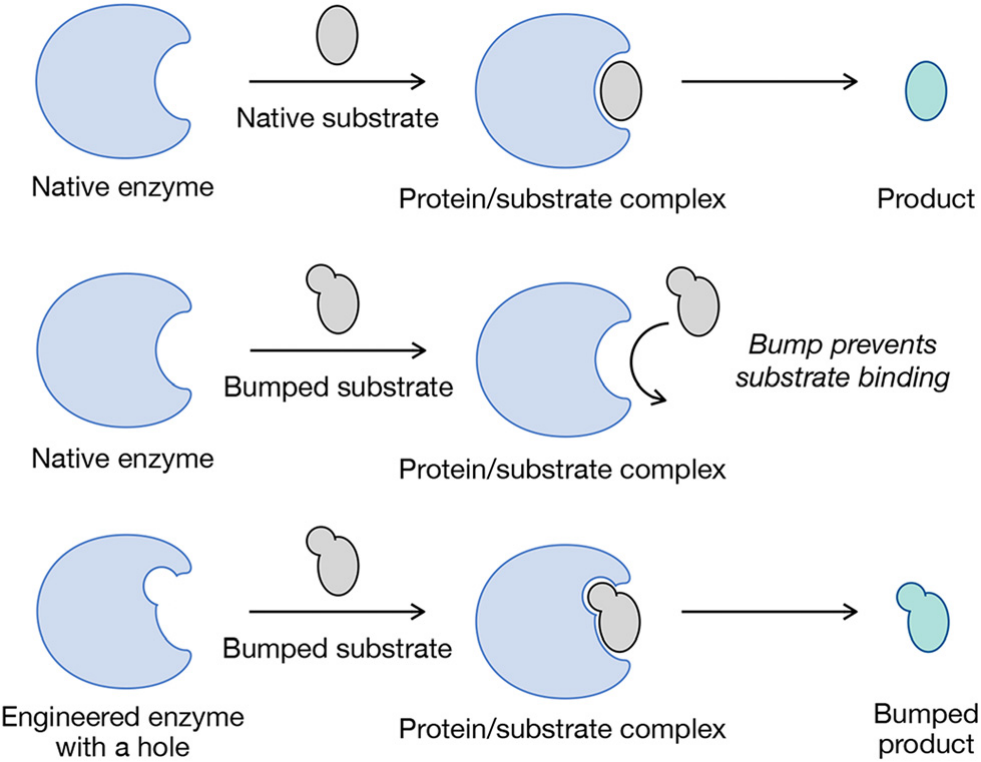
Chemical genetics through bump-and-hole. Native enzymes have active sites evolved to bind their native substrates. Engineering selectivity into enzymes can be accomplished by using chemistry to introduce a physical “bump” onto the substrate molecule. The bump prevents native enzyme from using the new substrate. Genetics can then be exploited to introduce a complementary “hole” into the enzyme active site that can accommodate the new substrate, yielding selectivity for the engineered substrate/enzyme pair.

**Fig. 5:**
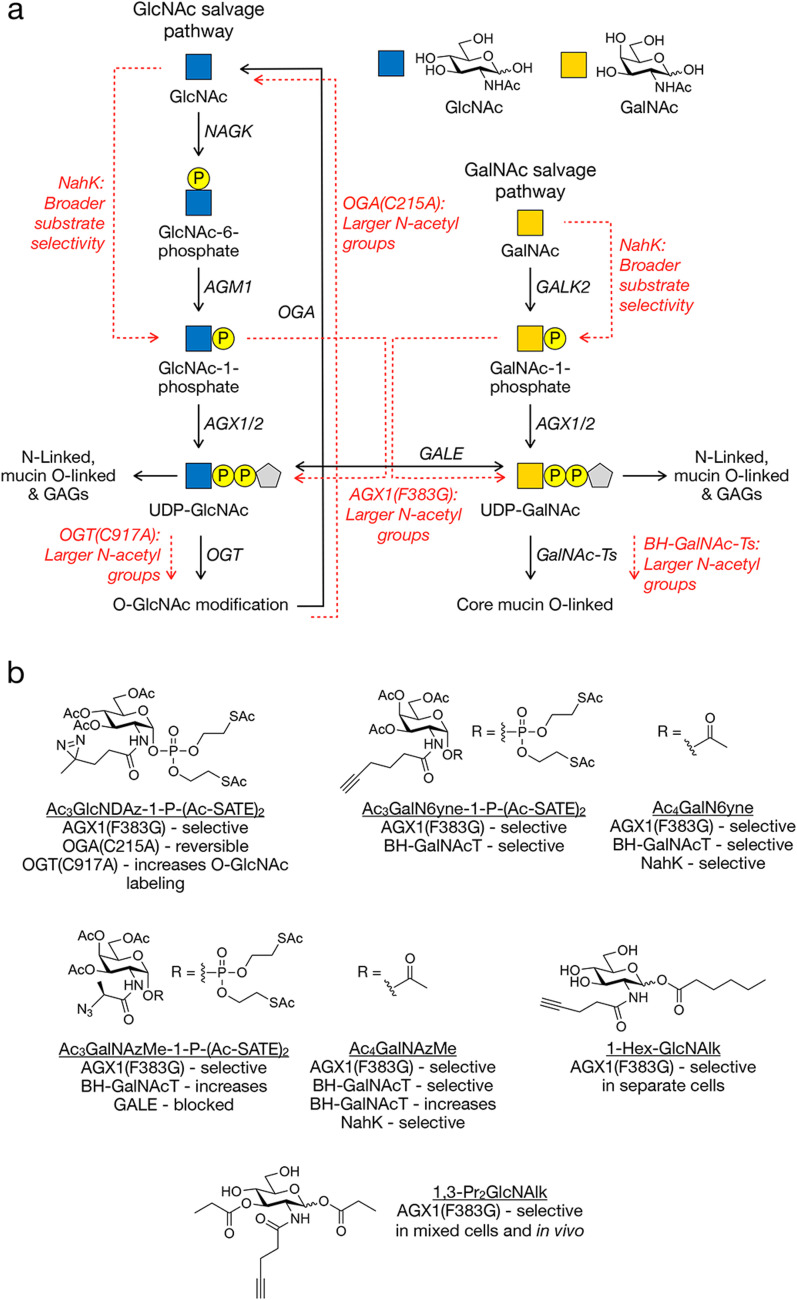
Monosaccharide pathways engineered for MCR selectivity. (a) Schematic of the GlcNAc and GalNAc salvage pathways (black) and enzymatic steps that have been engineered by bump-and-hole or bypass. (b) Examples of MCRs used in combination with the engineered metabolic pathways.

Traditional forward genetic screens – where random mutations are introduced, mutants with phenotypic defects are selected, and causative mutations are identified – have revealed the molecular underpinnings of many biological processes. However, this approach faces two key limitations: it is difficult to apply in mammals due to their slow reproduction and complex genomes, and most mutations are not conditional, as they cannot be controlled temporally. Nevertheless, forward genetics remains powerful for probing gene function within complex cellular environments. Chemical genetics, combining genetic engineering with small molecules, provides a conditional alternative by using small molecules to modulate protein function in living systems.^87–89^ Because ligands can be added or removed at will, this approach allows temporal control and is well suited for use in mammalian cells. Additionally, chemical genetic strategies can render certain cells or even enzymes sensitive to small molecules, while endogenous systems remain resistant. In recent years, chemical genetic tools have greatly expanded our capacity to dissect biological pathways and explore potential therapeutic targets. For example, as virtually all biological processes rely on transient protein–protein interactions, the rapamycin-inducible FKBP-FRB dimerization system demonstrated how ligand-induced interactions can control cellular processes with spatial and temporal precision.^90^ However, a persistent challenge in this field has been achieving selectivity, due to the conserved nature of ligand-binding sites. To overcome this, allele-specific chemical genetics – most notably the “bump-and-hole” approach – has emerged as a powerful solution (Fig. 4).^91^ By introducing complementary steric modifications to both ligand and target, researchers achieve orthogonal binding between engineered ligands and mutant proteins, with minimal cross-reactivity to wild-type counterparts. This strategy has been especially impactful in kinase biology, where enlarged ATP-binding pockets in mutant kinases are selectively targeted by sterically “bumped” inhibitors.^92,93^ The Shokat Lab has applied this method to uncover kinase substrates and functions across various systems, including whole organisms, expanding our understanding of kinase-mediated signaling in processes such as cell-cycle progression, transcription, and oncogenesis.^94,95^ Similarly, several labs have contributed to using bump-and-hole, as well as other strategies to engineer MCR metabolism and incorporation into glycans (Fig. 5(a)).

To our knowledge the Kohler lab was the first to successfully combine a bump-and-hole strategy with MCRs. Specifically, they were interested in identifying protein-interactions that are dependent upon O-GlcNAc modification in living cells and chose to use an GlcNAc-based MCR containing a diazirine photocrosslinker at the *N*-acetyl position, termed GlcNDAz.^96^ However, the large size of the diazirine group prevented this MCR from being efficiently metabolized to the corresponding GlcNDAz-1-phosphate (Fig. 5(b)). To address this, the authors synthesized a 1-phosphate analog (GlcNDAz-1-P(Ac-SATE_2_)), which passively diffuses across the plasma membrane and is deprotected by intracellular esterases. However, incorporation *via* the remainder of the native GlcNAc salvage pathway was inefficient, as the analog was not accepted by AGX1, the pyrophosphorylase that converts GlcNAc-1-phosphate into UDP-GlcNAc, the substrate for O-GlcNAc transferase (OGT). Structural analysis of AGX1 enabled the design of a mutant (F383G or F383A) with an expanded active site capable of accommodating the diazirine group and catalyzing the formation of UDP-GlcNDAz. This modified nucleotide sugar was then utilized by endogenous OGT to glycosylate proteins. Upon UV irradiation, the diazirine moiety formed covalent crosslinks with nearby proteins, allowing selective enrichment of O-GlcNAc-modified proteins and their binding partners. Proteins lacking the O-GlcNDAz modification were not cross-linked and thus excluded from detection in high-molecular-weight ranges by SDS-PAGE. This strategy enabled identification of substoichiometric interactors, including interactions between FG-repeat nucleoporins and nuclear import receptors such as TNPO1, supporting a role for O-GlcNAc in nuclear transport regulation.

One potential issue with GlcNDAz is that it cannot be removed from proteins by wild-type O-GlcNAcase (OGA), posing the risk of artificially stabilizing glycosylation and perturbing cellular processes. Given that OGA inhibition has been associated with cell cycle dysregulation and cytotoxicity, maintaining dynamic O-GlcNAc cycling is essential for physiological relevance. Although no overt toxicity was observed with O-GlcNDAz expression, subtle effects may go undetected by standard cell viability assays. Recognizing the importance of O-GlcNAc turnover, the Kohler lab identified OGA mutants capable of cleaving the modified sugar.^97^ Homology modeling based on bacterial glucosaminidases revealed steric hinderance in the OGA active site as a barrier to GlcNDAz hydrolysis. Among six active-site mutants tested, substitution of C215 with alanine or glycine restored hydrolytic activity, enabling dynamic O-GlcNAc cycling. The resulting cells that could cycle the O-GlcNDAz modification showed no effect on cell viability, validating the approach for reversible and physiologically relevant labeling of O-GlcNAcylated proteins in live-cell studies.

To improve the efficiency of O-GlcNDAz-mediated glycoprotein labeling, Kohler then engineered OGT to enhance its utilization of UDP-GlcNDAz, which is poorly accepted by the wild-type enzyme.^98^ Structure-guided mutagenesis identified a single point mutation, C917A, that relieved steric constraints and reversed substrate preference, improving catalytic efficiency with UDP-GlcNDAz fourfold compared to the wild-type enzyme with UDP-GlcNAc. This mutation enabled more effective glycosylation and photocrosslinking to O-GlcNAc-binding proteins, expanding the set of detectable O-GlcNAc-mediated interactions while maintaining glycosylation dynamics similar to the native system. Notably, OGT (C917A) also tolerated bulkier analogs at the *N*-acetyl position, suggesting broader applicability for future sugar analogs. When co-expressed with the engineered AGX1 and OGA variants, the system enabled both labeling and turnover of O-GlcNDAz-modified proteins in a cell-selective manner that mirrored wild-type O-GlcNAc dynamics.

Building upon this work the Bertozzi Lab sought to engineer individual glycosyltransferases of a larger family using the bump-and-hole strategy with the goal of deconvoluting their protein substrate preferences.^99^ Among the most prominent glycosyltransferase families is the *N*-acetylgalactosaminyltransferase (GalNAc-T) family, which initiates mucin-type O-GalNAc glycosylation on Ser/Thr residues. Although GalNAc-T expression is often associated with tumorigenesis, the lack of glycosylation consensus sequence and the diversity of glycan elaboration patterns hinder isoenzyme-specific functional studies. In an effort to circumnavigate this roadblock, the authors engineered mutant GalNAc-Ts (BH-GalNAc-Ts) that accept modified UDP-GalNAc analogs, like Ac_3_GalN6yne-1-P(Ac-SATE_2_) (Fig. 5(b)), not utilized by wild-type enzymes, while maintaining wild-type substrate-specificity and cellular localization. When combined with AGX1(F383G) mutants, which could synthesize the corresponding nucleotide sugars from synthetic sugar-1-phosphate precursors, this system allowed selective transfer of alkyne-containing GalNAc analogs onto native substrates. The bioorthogonal alkyne tag enabled downstream derivatization *via* click chemistry. This system preserved native glycan elaboration and overcame the limitations of gene knockouts and oversimplified *in vitro* model systems, providing unprecedented resolution for studying individual GalNAc-T function – a significant advance in metabolic glycan engineering.

A separate limitation of early GalNAc analogs was their susceptibility to UDP-glucose 4-epimerase (GALE)-mediated interconversion to UDP-GlcNAc analogs, resulting in nonspecific labeling. To overcome this, the Schumann lab developed a branched azide-containing GalNAc analog—GalNAzMe—with resistance to GALE-mediated epimerization.^100^ The caged sugar-1-phosphate precursor GalNAzMe-1-P(Ac-SATE_2_) (Fig. 5(b)) was efficiently biosynthesized in cells expressing mutant AGX1 and incorporated into mucin-type glycans, as confirmed *via* in-gel fluorescence, enzymatic assays, and mass spectrometry. This strategy enabled selective labeling of O-GalNAc glycans while avoiding off-target incorporation into GlcNAc-linked structures, without perturbing broader glycan metabolism by necessitating the knockout of GALE. Co-expression of BH-GalNAc-T2 enzyme further enhanced incorporation, demonstrating a level of programmable, isoform-specific labeling. Additionally, transposase-mediated transformation of both engineered AGX1 and GalNAc-T2 from a single vector improved delivery efficiency and made the approach suitable for diverse cell lines and model systems.^23^

Early studies of MCRs demonstrated that relatively small azide substituents on GlcNAc and GalNAc derivates are tolerated by native biosynthetic pathways, but the above studies suggested that intermediately sized groups, such as short alkynes, could be poorly metabolized. In fact, our group has previously shown that per-*O*-acetylated GlcNAc and GalNAc analogs bearing alkyne groups at the *N*-acetyl position (Ac_4_GlcNAlk and Ac_4_GalNAlk, respectively) were metabolized with low efficiency to their corresponding UDP-sugar forms in certain mammalian cells.^101^ Building on this finding, the Schumann lab investigated whether the same AGX1(F383G) mutant could also improve the utility of these MCRs.^102^ They found that labeling efficiency from Ac_4_GlcNAlk and Ac_4_GalNAlk increased significantly. In wild-type cells, the two MCRs labeled qualitatively similar proteins, while in GALE-knockout cells, Ac_4_GalNAlk and Ac_4_GlcNAlk labeled distinct glycoprotein subsets. Ac_4_GalNAlk showed predominant labeling of mucin-type O-glycoproteins, as confirmed by mucinase digestion. Compared to azide-based reagents, alkyne-tagged sugars produced narrower but more defined labeling profiles, suggesting that they showed reduced interconversion by GALE.

The introduction of per-*O*-acetylated sugar analogs significantly expanded the glycobiology toolkit, enabling researchers to incorporate small abiotic functional groups into glycans for participation in bioorthogonal reactions. This capability facilitates the tagging, identification, and visualization of glycoproteins in biologically relevant systems. As described above, the *O*-acetyl groups on MCRs mask the polar hydroxyls of monosaccharides, improving membrane permeability and allowing for robust labeling at relatively low concentrations. Once inside the cell, endogenous esterases remove the acetyl groups, releasing the free sugar to enter its respective salvage pathway. However, certain patterns of deacetylation were found to cause non-specific, non-enzymatic labeling of cysteine residues.^103^ The details of this process are discussed below in the limitations section, but it can lead to false identification of proteins as glycosylation. Our lab sought to address this issue by preparing MCRs based on GlcNAlk bearing a single longer ester at only the anomeric position, aiming to preserve the lipophilicity necessary for cell uptake while eliminating non-enzymatic cysteine modification.^104^ HeLa cells treated with these MCRs, like 1-Hex-GlcNAlk (Fig. 5(b)), showed low levels of that correlated with the length of the ester chain, suggesting effective cellular uptake while eliminating background labeling observed with per-*O*-acetylated sugars. Notably, the level of MCR incorporation increased dramatically upon expression of the AGX1 mutant used above, suggesting that this chemical genetic combination might be used for cell-selective labeling of glycoproteins.

Almost immediately after, the Chen lab successfully demonstrated this cell/tissue-selectivity in a system they called genetically encoded metabolic glycan labeling (GeMGL).^105^ This approach employs the same GlcNAlk/AGX1 bump-and-hole system with another MCR, 1,3-Pr_2_GlcNAlk (Fig. 5(b)), that displays low background cysteine modification. Using GeMGL, the authors achieved precise, cell-type-specific labeling in mixed cell co-cultures, tumor xenografts, and transgenic mice engineered to express the AGX1(F383G) mutant specifically in cardiomyocytes. Proteomic analysis revealed over 500 cardiomyocyte-specific O-GlcNAcylated proteins, many of which were enriched in mitochondrial and metabolic functions – underscoring the power of GeMGL for resolving cell-specific glycosylation *in vivo*.

The Shumann lab then combined the above observations and created BOCTAG (bio-orthogonal cell-type specific tagging of glycoproteins), a chemical genetic strategy that combined the metabolic engineering of the salvage pathway similar to GeMGL and combined it with BH-GalNAc-T mutants for transferase selectivity.^106^ The major advance of this system was the inclusion of the promiscuous bacterial kinase, NahK, that can generate the 1-phosphate-intermediates of a variety of MCRs that can then act as substrates for mutant AGX1. This extra level of engineering increases the range of MCR structures that can be exploited without the requirement for pre-synthesis of MCR-1-phosphates, which were previously required due to incompatibility of certain MCRs with upstream metabolic enzymes. This increased versatility was subsequently exploited by the same lab to introduce their MCR with a branched modification, GalNAzMe (Fig. 5(b)), again circumventing epimerization by GALE and allowing for selective labeling of mucin O-linked glycoproteins. Alternatively, the authors demonstrated that they could use the same system to metabolize Ac_4_GalN6yne (Fig. 5(b)), which can be directly combined with AGX1(F383G) and the BH-GalNAcTs without the pre-incorporation of the 1-phosphate.^107^ This dual-pathway accessibility expanded the range of metabolic reporters available for cell-selective glycoproteome analysis, enhancing the precision and flexibility of glycan profiling strategies.

Together, these advances highlight the rapid evolution of chemical genetic methods to enable increasing levels of MCR selectivity, driven by the biological information that would come from complete glycoproteome mapping. By combining structure-guided enzyme engineering with bioorthogonal chemistry and synthetic sugar analogs, researchers have achieved unprecedented precision in labeling specific glycosylation types, isoforms, and cell populations. These platforms now enable dynamic, cell-type-specific glycoproteomic analyses in complex biological models, paving the way for deeper insights into the functional roles of glycosylation in health and disease.

## Considerations and limitations

Despite the clear power and potential of MCRs for the cell-selective identification of glycoproteins, they are not without inherent limitations that should be considered during data interpretation. Because MCRs take advantage of the same enzymatic pathways as natural monosaccharides, they necessarily compete with these native metabolites.^30^ This competition has the potential to result in the buildup of both natural and MCR intermediates that can alter sensitive feedback mechanisms that control the relative abundance of different donor sugars. Additionally, addition of MCRs to the media likely results in increased flux through these biosynthetic pathways, possibly resulting in higher than “normal” levels of UDP-donors that in turn may yield more glycosylation than would normally be seen under native conditions. Additionally, the carbohydrate salvage pathways that MCRs rely upon have several points of interconnectedness, where one class of monosaccharide can be converted into another. For example, the enzyme UDP-galactose-4-epimerase (GALE) interconverts UDP-GalNAc and UDP-GlcNAc,^108^ and this enzyme can also convert some MCRs like GlcNAz shown in Fig. 1.^109^ This interconversion can be avoided in some cases through MCR engineering. For example, the ppGalNAcT-selective MCRs described above are largely not GALE substrates as the larger N-acetyl-group does not fit into the active site.^100,101^ Additionally, the enzymatic properties of different glycosyltransferases can result in the selective utilization of certain MCRs even if interconversion occurs. For example, several MCRs appear to be selectively transferred by the promiscuous O-GlcNAc transferase.^110–116^ However, researchers should generally view MCRs as discovery tools that can identify probable glycoproteins. Ideally, these initial hypotheses should be confirmed using direct methods like chemoenzymatic labeling or protein immunoprecipitation followed by mass spectrometry.

**Fig. 6:**
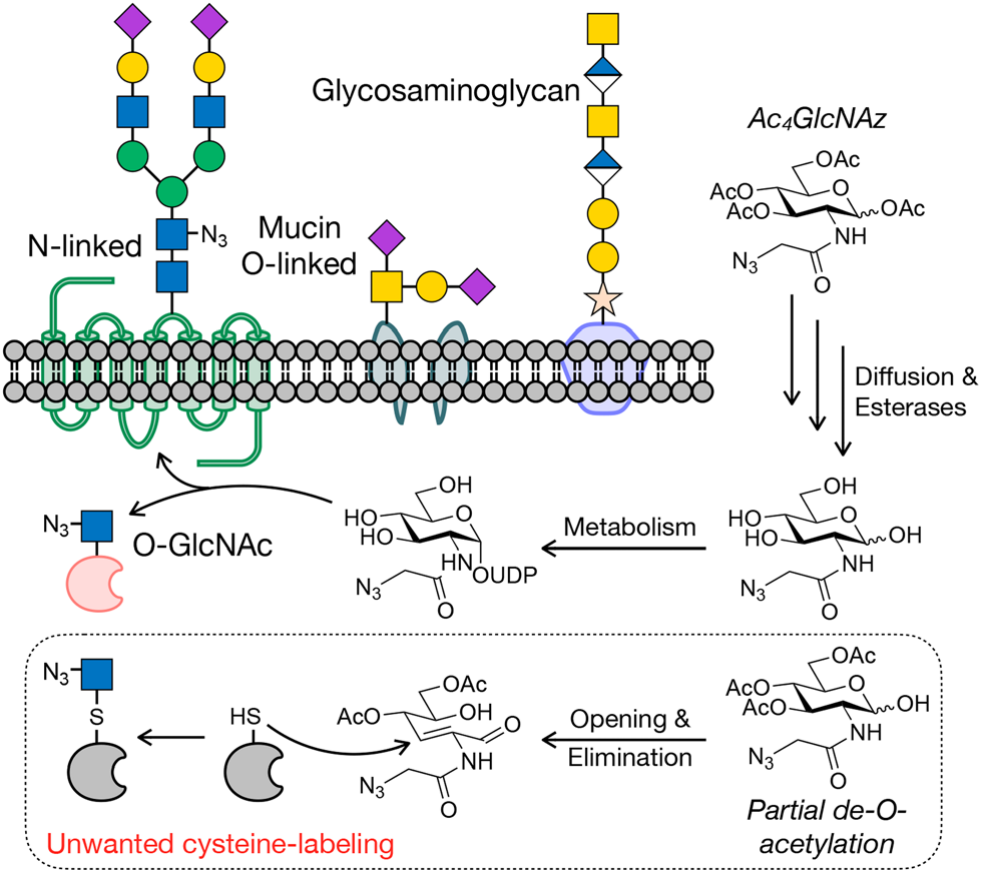
MCR incorporation into glycans is in competition with background cysteine labeling. If the 1-(anomeric) hydroxyl is de-esterified before others, the MCR sugar-ring can open revealing an aldehyde. Subsequent elimination of the 3-ester results in a Micheal acceptor that can react with cysteine residues, resulting in background chemical-labeling.

As mentioned briefly above, per-*O*-acetylated MCRs have the potential to modify sulfhydryl groups including cysteine side chains in proteins.^103^ This “off-target” labeling has the potential to lead to the potential enrichment and identification of proteins that are not glycosylated. Mechanistically (Fig. 6), this chemical modification occurs when the anomeric acetate of the MCR is removed before at least the *O*-acetate at the 3-position.^117^ This leads to the opening of the sugar ring and subsequent elimination of the 3-*O*-acetate resulting in an α,β-unsaturated aldehyde. This Michael acceptor can then react with cysteines. This pathway had gone unnoticed until 2018 for a couple of reasons. First, many of the initial MCR-characterization experiments focused on cell surface glycoproteins, where most cysteines are oxidized to disulfides and thus are non-nucleophilic. Second, this type of modification falls into the “you can’t see what you don’t look for” trap of traditional mass spectrometry, where most software looking to localize glycosylation onto peptides were likely focused on O- or N-linkages. Additionally, the chemical modification product has the same exact mass as a thioglycoside (S-linkage at the anomeric position), which O-GlcNAc transferase can make. Only after very careful analysis by the Chen lab, was the chemical nature of this background modification confirmed. The amount of this off-target labeling appears to range greatly (∼20–95%) depending on the MCR, concentration, cell type, *etc.*^103,114^ Strategies to avoid cysteine modification, some of which are included in the MCRs above, include limiting the number of O-esters to prevent elimination or incorporation of 1-phosphates that presumably largely stop the sugar ring from opening.^104,105,117,118^ While it has not been explicitly tested, we speculate that NahK expression in engineered cells may promote 1-phosphorylation, limiting the opening and elimination mechanism. However, cysteine modification is yet another reason to use direct methods to confirm protein glycosylation.

## Conclusions

Metabolic chemical reporters are key tools for identifying potential glycoproteins. For example, a cataloging of O-GlcNAc modified proteins in 2021 found that ∼80% of the potential glycoproteins were found using bioorthogonal chemistry.^119^ Although this number includes proteins identified using chemoenzymatic modification of O-GlcNAc residues,^120^ it demonstrates the power of MCRs. Biology fundamentally occurs in multicellular systems. The continued development of cell-selective MCR technologies has the potential to help unlock the functions and interplay of glycans in these systems. We envision applications both in living animals but also in increasingly complex organoid cultures, where specific expression of receptors or genetic engineering using selective promoters will drive incorporation of MCRs into certain cell/tissue types. Combined with increasingly sensitive mass spectrometers and agile software for searching possible glycan structures, we anticipate that MCRs will continue to play important roles in glycoscience discovery.

## Conflicts of interest

There are no conflicts to declare.

## Data Availability

No primary research results, software or code have been included and no new data were generated or analysed as part of this review.
